# PDM4, a Pentatricopeptide Repeat Protein, Affects Chloroplast Gene Expression and Chloroplast Development in *Arabidopsis thaliana*

**DOI:** 10.3389/fpls.2020.01198

**Published:** 2020-08-11

**Authors:** Xinwei Wang, Lirong Zhao, Yi Man, Xiaojuan Li, Li Wang, Jianwei Xiao

**Affiliations:** ^1^ Beijing Advanced Innovation Center for Tree Breeding by Molecular Design, Beijing Forestry University, Beijing, China; ^2^ College of Biological Sciences and Biotechnology, Beijing Forestry University, Beijing, China; ^3^ College of Horticulture and Plant Protection, Yangzhou University, Yangzhou, China

**Keywords:** pigment-defective mutant4, chloroplast, pentatricopeptide repeat protein, development, gene expression

## Abstract

Extensive studies have been carried out on chloroplast gene expression and chloroplast development; however, the regulatory mechanism is still largely unknown. Here, we characterized Pigment-Defective Mutant4 (PDM4), a P-type PPR protein localized in chloroplast. The *pdm4* mutant showed seedling-lethal and albino phenotype under heterotrophic growth conditions. Transmission electron microscopic analysis revealed that thylakoid structure was totally disrupted in *pdm4* mutant and eventually led to the breakdown of chloroplasts. The levels of several chloroplast- and nuclear-encoded proteins are strongly reduced in *pdm4* mutant. Besides, transcript profile analysis detected that, in *pdm4* mutant, the expression of plastid-encoded RNA polymerase-dependent genes was markedly affected, and deviant chloroplast rRNA processing was also observed. In addition, we found that PDM4 functions in the splicing of group II introns and may also be involved in the assembly of the 50S ribosomal particle. Our results demonstrate that PDM4 plays an important role in chloroplast gene expression and chloroplast development in Arabidopsis.

## Introduction

Chloroplasts are known for providing energy and carbon resource to the plant cell and are also indispensable for plant development and growth (Bryant et al., 2011). Derived from cyanobacterial ancestors, the chloroplasts belong to semi-autonomous organelles which possess their own genomes. Over the last billion years, the chloroplast genome has lost numerous genes in higher plants and generally remains about 120 genes which encode primary components of translation, transcription, and photosynthesis apparatus, as well as contains some critical biogenesis-related genes such as *accD*, *clpP1*, *matK*, *ycf1*, and *ycf2* (Sato et al., 1999; Leister, 2003; Ouyang et al., 2017). Although the chloroplast genome is small and with limited coding information, the transcriptional process is much more complex than that of prokaryotes which are usually organized in polycistronic transcriptional units. In particular, RNA processing from polycistronic precursors and editing are strikingly different between chloroplast and prokaryotes (Sugita and Sugiura, 1996; Sato et al., 1999).

Generally, in higher plant, two RNA polymerases with different origins participate in the transcription of plastid genes, including a plastid-encoded RNA polymerase (PEP) and nuclear-encoded RNA polymerases (Hajdukiewicz et al., 1997; Liere and Börner, 2007; NEP). During chloroplast development, the plastidic genetic system is first established within the proplastids. During this stage, the genes of PEP components and related ribosomal proteins were transcribed by NEP that is critical for the nascent construction of the plastid-genetic background. As a result, the activity of the transcriptional apparatus in the proplastid is remarkably raised. At the second stage, the well-assembled and functional PEP starts to transcribe plastid-encoded genes. Meanwhile, the photosynthetic proteins which are encoded by nuclear genes exhibit a high expression level and eventually lead to the establishment of photosynthesis systems (Mullet, 1993; Majeran et al., 2010; Tiller and Bock, 2014). PEP activity is also essential for the fully active chloroplasts formation because it promotes the expression of photosynthesis-related genes (Pfalz and Pfannschmidt, 2013). Subunits of the PEP core are present in two plastid protein preparations; one is associated with thylakoid and envelope membranes, and these are protein:DNA-complexes termed transcriptionally active chromosomes (TACs) (Krause and Krupinska, 2000; Pfalz et al., 2006; Krupinska et al., 2012). So far, the richest protein data set resulted from protein mass spectrometry analysis of isolated pTACs from Arabidopsis (*Arabidopsis thaliana*) and mustard (*Sinapis alba*), in which 35 proteins were identified (Pfalz et al., 2006). Eighteen of these proteins were denoted pTAC proteins, and three of them (pTAC2, -6, and -12) were shown to be required for plastid gene expression (Pfalz et al., 2006). Besides, PEP forms a complex with PEP-associated proteins (PAPs), and the *Arabidopsis thaliana* nuclear genome contains at least 12 PAP genes (Yu et al., 2014), and all PAPs have also been identified in the nucleoid or TAC proteomes (Pfalz et al., 2006; Majeran et al., 2012; Melonek et al., 2016).

The complexity of the chloroplast gene expression system is also highly regulated at the post-transcriptional which mainly reflects the extensive modiﬁcations exerted on transcripts during RNA processing (Chi et al., 2015). For instance, defects in endonucleolytic cleavage polycistronic transcripts would result in a blocking translation in chloroplast mRNAs (Sugiura et al., 1998; Walter et al., 2010). And critical sites of chloroplast RNAs that are essential for chloroplasts development in higher plant can be correctly spliced or edited (Bobik et al., 2017; Du et al., 2017; Zhang J. et al., 2017).

Chloroplast RNA metabolism refers to a substantial number of RNA-binding proteins (Stern et al., 2010). Due to the limited coding capacity of the chloroplast genome, the chloroplast gene expression is controlled both by plastid-encoded and nucleus-encoded proteins (Germain et al., 2013; Stoppel and Meurer, 2013; Belcher et al., 2015). As nucleus-encoded factors, it has been demonstrated that the pentatricopeptide repeat (PPR) proteins participate in chloroplast gene expression and function (Barkan and Small, 2014). Members of the PPR protein family are considerably numerous in land plants with up to 450 representatives in Arabidopsis (Lurin et al., 2004), and this family is characterized by PPR repeat with highly degenerate unit of 35 amino acids (Lurin et al., 2004). In addition, according to the variation in length and amino acid composition of PPR repeats, the PPR proteins have been used to define two categories; P-class PPR proteins are mainly composed of typical 35-amino acid sequence repeats, and the PLS-class members contain triplets of motifs by varied amino acid lengths and sometimes with an additional C-terminal domain (Lurin et al., 2004; Cheng et al., 2016).

The PPR proteins can directly bind to chloroplast RNAs and prevent targeted RNAs from RNase degradation and/or facilitate or directly participate in related processing (Small and Peeters, 2000; Pogson and Albrecht, 2011). It is widely accepted that the PLS subgroup proteins are mostly involved in RNA editing, whereas the P subgroup proteins play crucial roles in intron splicing, RNA stabilization, and translation process (Barkan and Small, 2014). For example, either PPR protein DYW2 or NUWA can be interacted with CLB19, function in editing of organelle RNA (Guillaumot et al., 2017). Seedling Lethal1 (SEL1/PDM1), a PPR protein was proved to participate in the plastid gene expression and chloroplast development at an early stage (Pyo et al., 2013). Through specifically recognizing RNA sequence of 23S–4.5S rRNA precursor, PPR protein SOT1 performs the endonucleolytic activity during the maturation of 23S and 4.5S rRNA in chloroplast (Zhou et al., 2017). SOT5/EMB2279 is involved in intron splicing of plastid *rpl2*, and PpPPR_66 acts as a processing factor to assist *ndhA* pre-mRNA splicing by bounding preferentially to the specific region (Huang et al., 2018; Ito et al., 2018). Besides, some PPR proteins are proved to be required for the accumulation/assembly of plastid ribosomes (Williams and Barkan, 2004). Synthesis of the rRNAs and proteins, with correct folding, maturation/modification, and further assembly into functional particles, are highly coordinated. The 30S subunit includes 16S rRNA and about 20 ribosomal proteins, while the 50S subunit consists of 23S, 5S, and 4.5S rRNAs and about 30 ribosomal proteins (Yamaguchi et al., 2003).

In this study, we report the *pdm4* mutant of Arabidopsis, which was generated by T-DNA insertion and screened as defective pigment and seedling-lethal phenotype. The corresponding gene, *PDM4*, encodes a putative chloroplast PPR protein. Subsequent genetic and molecular analyses suggest that PDM4 is involved in the regulation of plastid gene expression and chloroplast development.

## Materials and Methods

### Plant Materials and Growth Conditions

*Arabidopsis thaliana* Columbia (Col-0) ecotype and the mutant *pdm4* (SALK_034168, obtained from the Arabidopsis Biological Resource Center; ecotype Columbia) were used in all experiments. The seeds were sterilized by 0.8% NaClO for 10 min, followed by 5 times rinsing with sterile double-distilled water. Then they were plated on 1/2 MS medium with 1% sucrose and 0.8% agar (pH 6.0) at 4°C in the dark for 48 h after sterilizing. Next, they were transferred to long-day conditions (16 h light, 8 h dark) at 22°C. The T-DNA insertion was confirmed by PCR analysis and subsequent sequencing with the primers LBb1.3 (5′-ATTTTGCCGATTTCGGAAC-3′) and *pdm4*-RP. The homozygous *pdm4* mutant line was verified by PCR using specific primers *pdm4*-LP 5′-TCACTAACCAATAACACCACC-3′ and *pdm4*-RP 5′-ATTGCTTGTGAGCCTTGGT-3′.

### Total RNA Isolation and Reverse Transcription (RT)-PCR; Quantitative Real-Time PCR (qRT-PCR) Analysis

Three total RNA samples were extracted from light grown 3-week-old mutant and wild-type plants with RNeasy Mini Kit (Tiangen Biotech Company, Beijing, China). For RT-PCR analysis, first strand cDNA was synthesized by the one-step reverse-transcription system (TransGen Biotech, Beijing, China), and the operation was carried out based on previous protocol (Zhang J. et al., 2017). The qRT-PCR was performed, and the primers used in this analysis were according to Du et al. (2017). All the measurement for each sample was repeated three times.

### Measurement of Chlorophyll

For measuring the chlorophyll content, leaves from 3-week-old Arabidopsis seedlings were collected. One hundred milligram of leaves was ground in liquid nitrogen into fine powder and mixed thoroughly with 250 μl 80% acetone and quantified on a UV2800 spectrophotometer (Unico Instruments Co., Ltd, USA). We calculated the chlorophyll content from the absorbance following the method of Arnon (1949). Three biological replicates, each with three repeats, were analyzed for each sample.

### Optical and Transmission Electron Microscopy

To observe the development of the embryos between wild-type and the mutant plants, the seeds of heterozygous *pdm4* mutant line were removed from siliques and totally cleared in Hoyer’s buffer (chloral hydrate, 50 g; gum Arabic, 3.75 g; and glycerol, 2.5 ml were dissolved in 15 ml of water) according to Meinke et al. (1994). The individual embryo in the cleared seed was examined by light microscopy (Zeiss, Axioskop, Germany) using differential interference contrast (DIC; Du et al., 2017). For transmission electron microscopy (TEM) analysis, the samples were first cut into ultra-thin pieces (LKB-8800, LKB, Broma, Sweden) and stained with alkaline lead citrate and uranyl acetate and then examined with a transmission electron microscope (JEM 1200EX, JEOL, Japan).

### Northern-Blot Analysis

For northern-blot analysis, total RNA from wild-type and *pdm4* seedlings was extracted and determined by using thermo NanoDrop 2000 (Thermo, USA). Three equal content RNA samples of the wild type and *pdm4* mutant were separated on 1.3% (w/v) agarose-formaldehyde gels and subsequently blotted to a nylon membrane. Next, the membrane was hybridized with a specific probe labeled with ^32^P. The labeled probes were obtained by using the Prime-a-Gene Labeling Kit (SGMB01-Promega-U1100, USA). The sequences of the primers were according to Du et al. (2017). All the analysis was performed at least in three independent repeats.

### Subcellular Localization of Green Fluorescent Protein

In order to study the subcellular localization of PDM4, two-week-old complemented Arabidopsis seedlings (*COM*) were cut into small pieces and incubated in a solution (10 mM MES, 20 mM CaCl_2_, 0.5 M mannitol, pH 5.8, containing 0.1 g ml^−1^ macerozyme and 0.1 g ml^−1^ cellulase) for 4 h at room temperature in the dark. Protoplasts were then isolated according to Dovzhenko et al. (2003) and assessed for fusion gene expression with a confocal microscope (TCS SP5 CLSM; Leica). The signal of green fluorescence was detected, and red fluorescence represents the auto-fluorescence of chlorophyll.

### Protein Extraction and Western Blot Analyses

The total proteins were extracted from 3-week-old wild type and *pdm4* mutant with NB1 buffer (pH 8.0), including 1 mM MgCl_2_, 5 mM DTT, 0.5 M sucrose, 50 mM Tris MES, 10 mM EDTA, and protease inhibitor cocktail. The different protein samples were separated by 15% SDS-page and then transferred onto PVDF membranes. And then, membranes were incubated with specific primary antibodies. The antibodies were used in this study according to Xiao et al. (2012). Signals were detected using enhanced chemiluminescence method (Du et al., 2017), and signal intensity of protein band was analyzed by “ImageJ” software. These experiments were repeated at least three times independently.

### Chloroplast Isolation and Fractionation

Chloroplast isolation method was performed as described previously with minor modifications (Aronsson and Jarvis, 2002). Exactly, 21-day-old Arabidopsis plants were ground in extraction buffer (5 mM EGTA, 50 mM HEPES-KOH pH 8.05, 0.33 M sorbitol, 5 mM EDTA, 10 mM NaHCO_3_, and 5 mM MgCl_2_). After filtering through Miracloth, the sample was centrifuged for 1 min at 1,000 g. The supernatants were removed, and pellets were re-suspended and then loaded onto Percoll gradients (70 and 40% in isolated buffer respectively); then intact chloroplasts were collected and washed three times with washing buffer (3 mM MgSO_4_, 0.33 M sorbitol, and 50 mM HEPES-KOH, pH 8.0). Chloroplasts were fractionated into the thylakoid membrane, stromal and envelope fractions as described by Du et al. (2017).

### RNA Immunoprecipitation Assays

The procedures used for coimmunoprecipitation and immunoblot assays were described previously (Terzi and Simpson, 2009) using 3-week-old *35S:PDM4-GFP* complemented seedlings. Anti-GFP magnetic beads were obtained from Abcam company (ab290, http://www.abcam.com/). The sequences of primers used to detect RNA content that coimmunoprecipitated with PDM4-GFP are listed in **Supplemental Table 1**.

## Results

### Characterization of the *pdm4* Mutant

**Figure 1:**
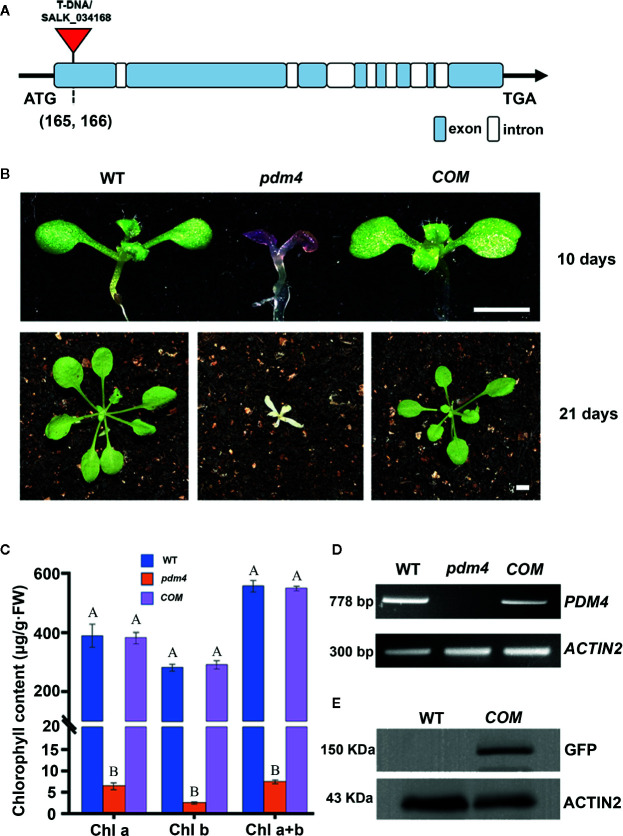
Identification and characterization of the *pdm4* mutant. **(A)** Gene structure of the *PDM4* (AT5G27270). Exons indicated by the wathet boxes, introns by the white boxes, and the T-DNA insertion indicated by the red triangle; ATG represents the initiation codon, and TGA represents the stop codon. **(B)** Pigment-defective phenotypes and complementation of the *pdm4* mutant. The cDNA of the *PDM4* was cloned into a binary expression vector with the GFP tag and complementation of the *pdm4* mutant (*COM*). WT, wild type. 10-day-old plants were grown on sucrose-supplemented medium (up lane), and 21-day-old plants were grown in soil (down lane). Scale bar: 3 mm. **(C)** The chlorophyll content of wild-type, *pdm4* and *COM*. Chlorophyll was extracted from 14-day-old seedlings and quantified. Values given are μg/g fresh weight ± SD (n = 3). Values not connected by the same letters are significantly different (Student’s t test, p < 0.05). The average of three replicates is shown. **(D)** Reverse transcription PCR analysis. RT-PCR was performed using specific primers for *AT5G27270* or *ACTIN2*. **(E)** Total proteins from wild-type and *COM* (15 μg) were separated by SDS-PAGE, followed by immunoblot analysis with the anti-GFP. The experiments of **(D, E)** were repeated three times independently.

To identify PPR genes involved in chloroplast development, we screened a collection of T-DNA inserted mutation lines localized in *PPR* genes (Du et al., 2017; Zhang J. et al., 2017). In this study, we obtained a new mutant, designated as *pigment-defective mutant4* (*pdm4*). The position of T-DNA insertion in *pdm4* was confirmed by PCR and subsequent sequencing, and the result exhibited T-DNA inserted in 165 base pairs downstream of putative start codon (**Figure 1A**).

When cultivated on 1/2 MS medium supplemented with 1% sucrose, the *pdm4* mutant had developed purple cotyledons that were gradually bleached to white with extended growth period (**Figure 1B**). After being transferred into the soil, the *pdm4* mutant was unable to grow and died shortly (**Figure 1B**). Homozygous *pdm4* plants are kept by segregating from a self-pollinated heterozygous plant with a ratio 3:1 in green and albino plants (data not shown). Thus, the albino phenotype is inherited as a recessive mutation. To confirm that the disruption of *PDM4* results in the lethal phenotype, we performed the functional complementation analysis. The result indicated that full-length coding region of *PDM4* gene fused a GFP tag at its C terminus successfully rescued the *pdm4* phenotypes. Among the 56 T1 transgenic lines analyzed, 16 lines were homozygous *pdm4* plants and showed a wild-type looking phenotype (**Figure 1B**).

The concentration of chlorophyll (μg/g FW) in the *pdm4* was significantly reduced compared with the wild type (**Figure 1C**). As expected, chlorophyll accumulated in *COM* plants was equivalent to the level of the wild-type plants. By reverse transcription-PCR analysis, obvious signals were obtained from *COM* and wild-type plants but not observed in homozygous *pdm4* mutant; this result demonstrated the expression of *PDM4* was completely suppressed (**Figure 1D**). Eventually, western blot result showed that PDM4-GFP proteins were located at about 150 kilodaltons (kDa) in complemented lines by using a GFP antibody, which is in accordance with the predicted protein molecular weight of GFP-tagged PDM4 (**Figure 1E**). The complementation analysis of the *pdm4* phenotype indicated that the PDM4-GFP is a functional protein, and *PDM4* gene was responsible for the phenotype of the *pdm4*.

### Chloroplast Development and Accumulation of Photosynthetic Proteins in *pdm4*

**Figure 2:**
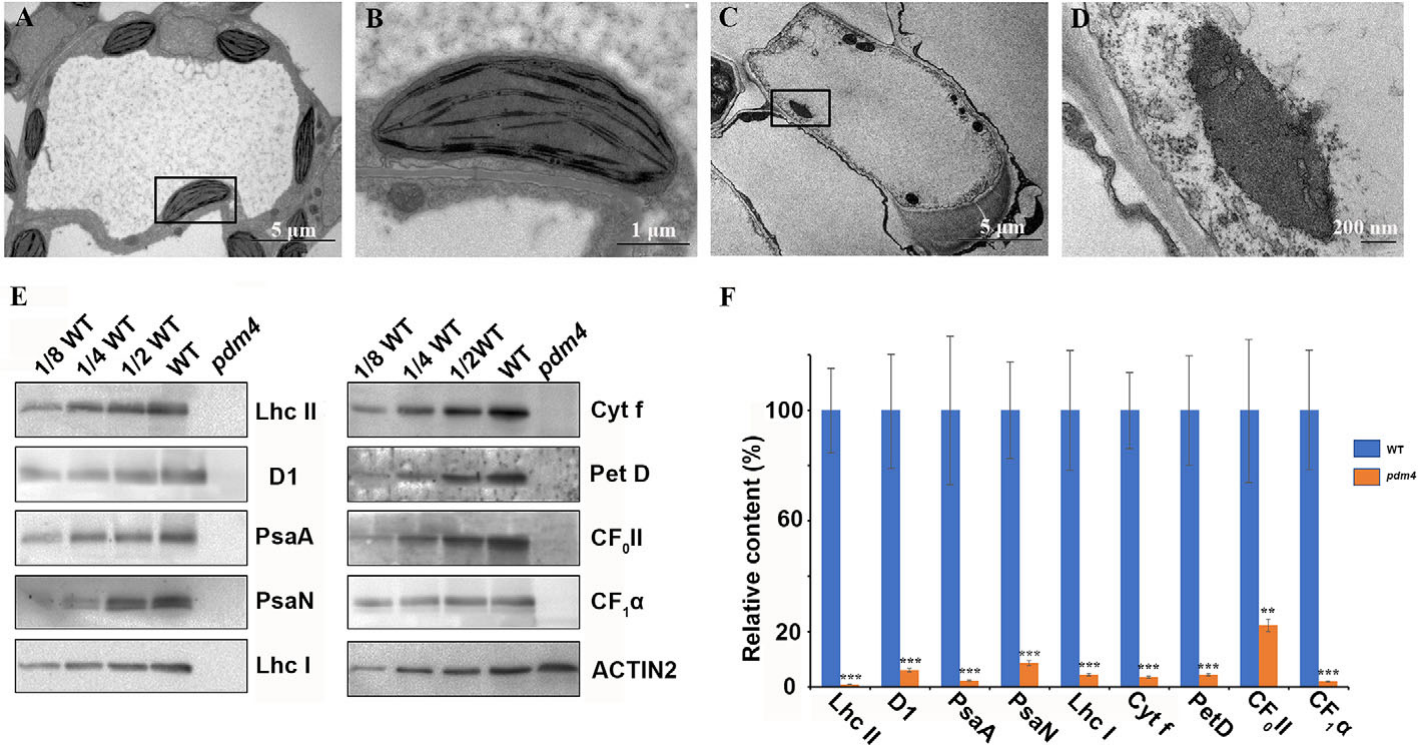
Ultrastructure of plastids and chloroplast proteins in *pdm4*. **(A–D)** The thylakoid membrane organization in the chloroplasts of wild type **(A, B)** and *pdm4* **(C, D)**. Scale bars: 5 μm in **(A)** and **(C)**; 1 μm in **(B)**, and 500 nm in **(D)**. **(E)** Accumulation of representative subunits of photosynthetic protein complexes determined by western blot analysis with specific antibodies. Total proteins from wild-type and *pdm4* seedlings were extracted and separated by 15% SDS-PAGE. Probes used specific anti-Lhc II, anti-D1, anti-PsaA, anti-PsaN, anti-Lhc I, anti-Cyt f, anti-Pet D, anti-CF0 II, anti-CF1*α*, and anti-ACTIN antibodies. **(F)** Semi-quantitative analysis of chloroplast proteins. After immunoblot analysis, the average signal intensities for each protein were quantified by the ImageJ software for three independent times. The protein relative contents (per unit of total protein) were determined and compared. Error bars represent standard errors. The relative protein level of *pdm4* mutants was obtained when protein level of wild type was set to 100. Similar result to that presented in **(E)** was obtained from three independent experiments. Results from a representative experiment are shown. The asterisks indicate significant differences between WT and *pdm4* (Student’s t test; **p < 0.01; ***p < 0.001).

Considering that most photosynthetic pigment defects may result in a retarded chloroplast development, we assessed the possibility that the *pdm4* mutation causes ultrastructural changes in the chloroplasts, and plastids from 3-week-old seedling mesophyll cells were examined by transmission electron microscopy (**Figures 2A–D**). The chloroplasts from wild type contained well-developed membrane systems featured with typical grana structure connected by the stroma lamellae, and the stroma thylakoid and grana thylakoid were easily distinguished (**Figures 2A, B**). Relative to wild-type chloroplasts, the *pdm4* plastids are smaller, deformed, and devoid of thylakoid membrane and granal stacks, and meanwhile, the membrane spacing was not clear (**Figures 2C, D**).

To obtain more information concerning the molecular lesion in *pdm4* to further explain the defects of chloroplasts development in corresponding mutant, total proteins were isolated, and equal sample volumes were loaded in denaturing polyacrylamide gel electrophoresis. Then we performed western blot analysis to detect accumulation of core subunits of photosynthetic complexes, including D1 and LHC II of PSII (encoded by *psbA* and *lhcb2*), PsaA, PsaN, and LHC I of PSI (encoded by *psaA, psaN*, and *Lhca1*), Cyt f and petD, the subunits of Cyt b_6_/f, and the CF1α, CF0 II of the ATP synthase. All these proteins were markedly reduced, even barely detected in *pdm4* mutant (**Figures 2E, F**). So, due to the dramatically decreased of some representative subunits of photosynthetic complexes in *pdm4* mutant, we got a conclusion that PDM4 is essential for the normal accumulation of thylakoid proteins.

### Homozygous *pdm4* Embryos Show Delay in Embryogenesis

**Figure 3:**
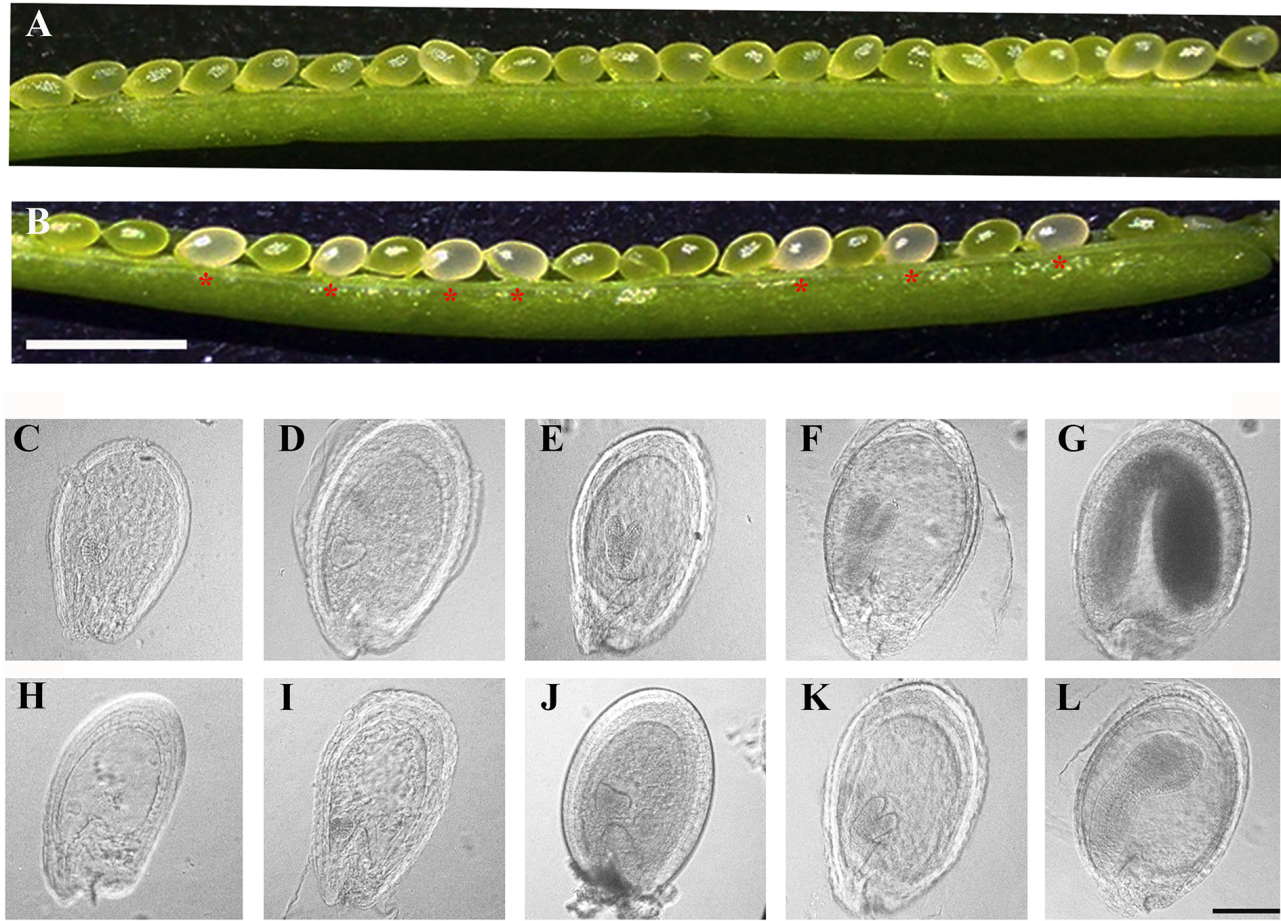
Phenotypic analysis in siliques and embryogenesis in *pdm4* mutant. **(A)** Seeds in wild-type plants. **(B)** Seed segregation in siliques from a *pdm4*/+ heterozygous plants. **(C–L)** Cleared seeds observed under differential interference contrast (DIC). Embryo development of wild type **(C–G)** and *pdm4* **(H–L)** at globular, heart, torpedo, walking-stick, and cotyledon stages. Scale bars: **(A, B)**, 2 mm; **(C–L)**, 1 mm.

To reveal the defects in *pdm4* development and embryos, we examined developing seeds at various developmental stages by using the differential interference contrast (DIC) optics. Within the immature siliques of heterozygous *pdm4*, the segregation ratio of the green and white seeds was close to 3:1 (data not shown) and consistent with the segregation in albino phenotype (**Figures 3A, B**). Moreover, assessment of cleared seeds from the same heterozygous silique indicated that the wild-type and heterozygous seeds underwent normal developmental stages (**Figures 3C–G**), whereas embryo development of homozygous seeds was seriously disrupted (**Figures 3H–L**). Different developmental stages of the seeds in wild-type were well-defined and in normal condition, and no difference was visible in *pdm4* compared with wild-type embryos before or at the early globular stage (**Figure S1**). But from the late globular stage to the early heart stage, developmental deviation of mutant embryos became apparent (**Figures 3C–L**).

### *PDM4* Encodes a Novel P-Type PPR Protein Localized in the Chloroplast

**Figure 4:**
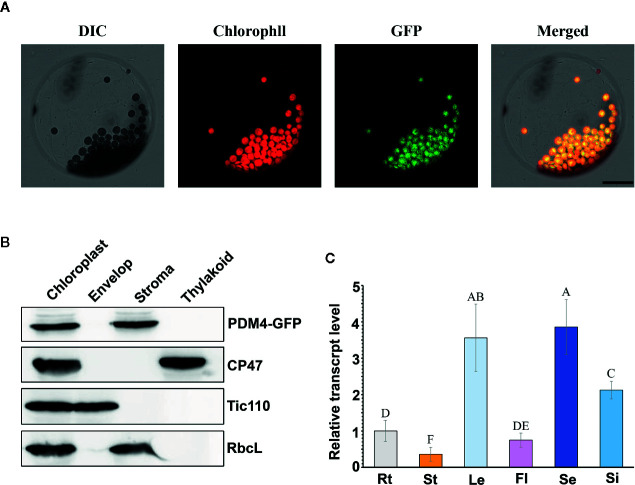
PDM4 protein location and gene expression pattern. **(A)** Localization of the PDM4 protein within the chloroplast using the GFP assay by protoplasts of complement line. Scale bars: 25 nm. **(B)** Immunolocalization of PDM4. Intact chloroplasts were isolated from the leaves of GFP-tagged complemented Arabidopsis, and then separated into envelop, stroma, and thylakoid membrane fractions. Antisera were used against GFP, the integral thylakoid membrane protein CP47, the integral inner envelop membrane protein Tic110, and abundant stroma protein RbcL. **(C)** Quantitative real-time (qRT)-PCR analysis of the *PDM4* gene in the root (Rt), stem (St), leaf (Le), flower (Fl), seedling (Se), and silique (Si). Values not connected by the same letters are significantly different (Student’s t test, p < 0.05). All the experiments were repeated three times independently.

Based on the redefined PPR motif, PDM4 is classified P-type PPR protein and possesses 16 PPR repeat domains (**Figure S2**). Sequence analysis of the *PDM4* gene revealed that it contains eight exons with a 3,114 base pair open reading frame, which encodes a polypeptide of 1,038 amino acids. By Chlorop1.1 software prediction, PDM4 possesses a putative 31 amino acid transit peptide at the N terminus. To investigate the subcellular distribution of PDM4, we extracted and observed the protoplasts from *COM* plants, and the results showed that the fusion proteins were exclusive to chloroplasts and colocalized with chlorophyll (**Figure 4A**). To further confirm the sublocalization of PDM4 within chloroplasts, intact chloroplasts from *COM* plants were isolated and further fractionated into stroma, envelop, and thylakoid membrane. **Figure 4B** shows that the GFP-tagged PDM4 protein is mainly located in the chloroplast stroma, but not in the thylakoid or envelope membrane fraction. Furthermore, qRT-PCR assay demonstrated that *PDM4* is universally transcribed throughout the various developmental stages; especially, a high expression level in the seedling and leaf and relatively low expression in the flower, stem, and root are observed (**Figure 4C**).

To identify PDM4 homologs in several species, a search was carried out using NCBI protein database and the protein sequence of PDM4 as the template. High similarity of protein sequences was selected for bioinformatics analysis. The PDM4 homologous proteins exhibited a relatively high level of similarity in *Populus trichocarpa* (Potri.T071500, 58.0%) and *Glycine max* (Glyma.08G106500, 54.8%), while they showed a low level in *Volvox carteri* (Vocar.0001s1331, 23.4%) (**Figure S3**). To further reveal the relationship between the PDM4 and other family member proteins in different species, we constructed a phylogenetic tree (www.phylogeny.fr) of the closest amino acid sequences from **Figure S3**, and analysis revealed that *PDM4*-like proteins are present in most chloroplastida including monocots, dicots, ferns, mosses, and algae (**Figure S4**).

### The *PDM4* Mutation Affects Plastid Gene Expression

Growing mass of information supports the idea that the PPR proteins are always involved in the regulation of plastid gene expression (Barkan and Small, 2014). Depending on the different RNA polymerases required, plastid genes can be roughly divided into three categories: genes of class I are mainly synthesized by PEP, genes of class II are synthesized by both PEP and NEP, and genes of class III are synthesized by NEP. To assess the possibility that PDM4 functions in gene expression in plastid, we examined the transcript levels of three types of genes both in the *pdm4* mutant and wild-type plant by qRT-PCR analysis.

**Figure 5:**
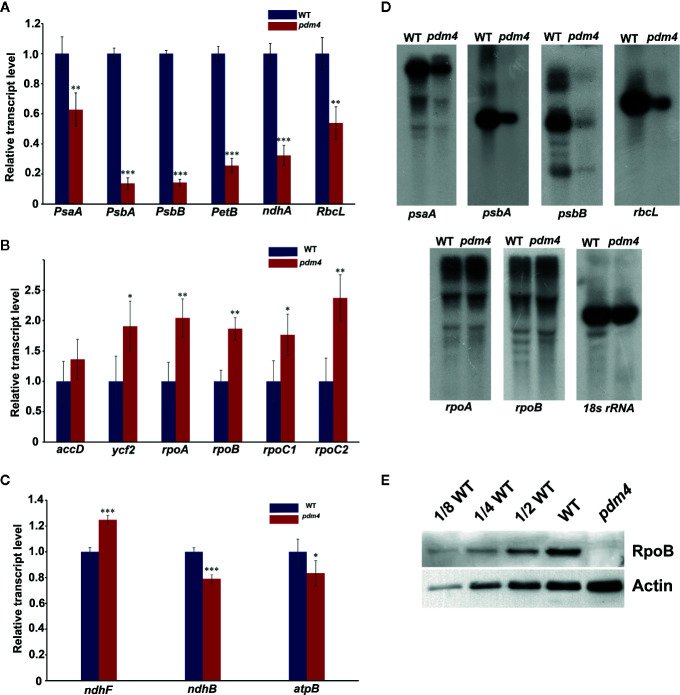
Chloroplast gene expression in *pdm4* relative to the wild type. **(A)** The expression levels of plastid-encoded polymerase (PEP)-dependent chloroplast genes. **(B)** The expression levels of nuclear-encoded polymerase (NEP)-dependent chloroplast genes. **(C)** The expression levels of both PEP- and NEP-dependent chloroplast genes. The asterisks indicate significant differences between WT and *pdm4* (Student’s t test; *p < 0.05; **p < 0.01; ***p < 0.001). **(D)** RNA gel-blot hybridization analysis of transcript levels for the different chloroplast gene classes. 5 μg of total RNA from 3-week old plants was size fractionated by agarose gel electrophoresis, transferred to a nylon membrane, and probed with ^32^P-labeled cDNA probes for *psaA*, *psbA*, *psbB*, *rbcL*, *rpoA*, *rpoB*, and *18S rRNA*. **(E)** RpoB protein content was indicated by western blot. Total protein (50 μg or the indicated dilution of the wild-type sample) from 3-week-old seedlings was loaded per lane. Actin was used as a loading control. These experiments obtained similar results each time. Results from a representative experiment of three times are shown.

We chose the six genes as representation of PEP-dependent (class I) type, *psaA*, *psbA*, *psbB*, *petB*, *ndhA*, and *rbcL*. The results indicated that the transcription level of PEP-dependent genes in *pdm4* was dramatically decreased from about 40 to 90% (**Figure 5A**). In contrast, the transcript levels of the plastid genes, which were tested as the representatives of class III (NEP-dependent) type, including *rpoA*, *accD*, *rpoB*, *ycf2*, *rpoC1*, and *rpoC2*, were all increased by varying degrees in *pdm4* (**Figure 5B**). As for the class II genes, synthesized by both NEP and PEP, *ndhB*, *atpB*, and *ndhF* expression levels showed a reverse trend. The transcript levels of *ndhB* and *atpB* decreased by nearly 20%, while the transcripts of *ndhF* were obviously upregulated (**Figure 5C**). The contents of representative genes were also confirmed by northern-blot analysis and the result is comparable to the qRT-PCR analysis (**Figure 5D**). To further study whether the increased transcription level of *rpoB* resulted in an enhancement at protein level, we detected the RpoB protein content in the *pdm4* mutant by western blot analysis. Compared with wild type, the result showed an obviously reduced level of RpoB in the *pdm4* mutant (**Figure 5E**).

### *PDM4* Involved in RNA Splicing of Multiple Chloroplast Group II Introns

**Figure 6:**
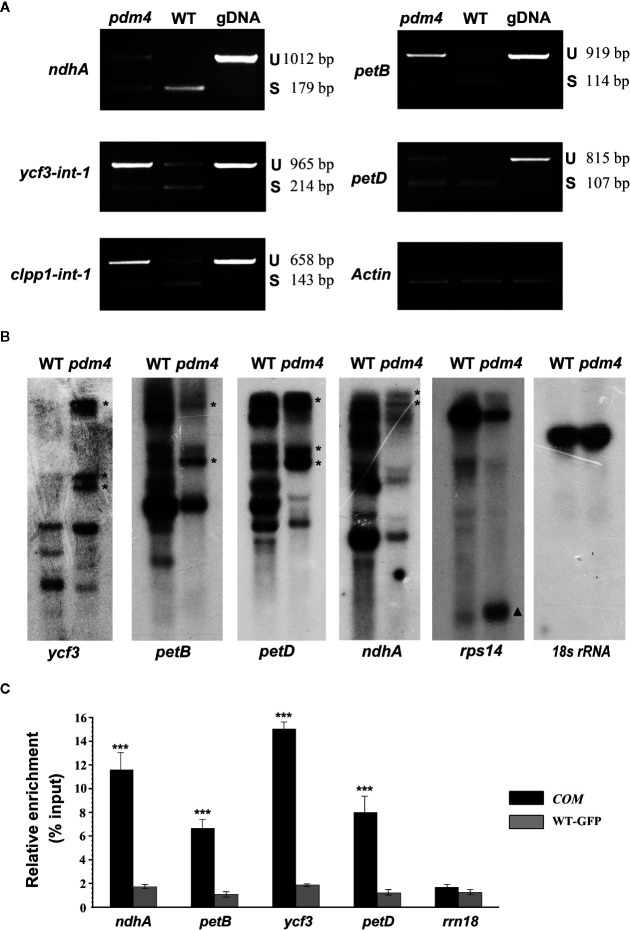
PDM4 is involved in RNA splicing of Arabidopsis group II introns. **(A)** Inefficient splicing of group II introns of *ndhA*, *petB*, *ycf3-int-1*, *petD*, and *clpp1-int-1* in the mutant as determined by reverse-transcription (RT)-PCR. S, introns are spliced; U, introns are retained. **(B)** Northern-blot analysis for the transcript accumulation and pattern of *ycf3*, *petB*, *petD*, *ndhA*, *rps14*, and *18S rRNA*. 5 μg of total RNA from 3-week-old seedlings was analyzed by hybridization to specific probes for each gene. Gene precursor indicated by the asterisk. The results shown are representatives of three independent biological replicates. **(C)** RNA immunoprecipitation analysis followed by a quantitative PCR assay. 3-week-old 35S:PDM4-GFP complemented seedlings were used. IP+, anti-GFP immunoprecipitation; IP−, mock immunoprecipitation. The asterisks indicate significant differences between WT-GFP and *COM* (Student’s t test; ***p < 0.001). Data are means (± SE) obtained from three replicates.

Dozens of P-type PPR proteins have been reported to function in RNA splicing in chloroplast (Beick et al., 2008; Barkan and Small, 2014). To determine whether PDM4 influences the splicing of the group II introns, representative splicing event was assayed in the *pdm4* mutant by performing RT-PCR analysis (**Figure 6A**). Compared with the wild-type plants, the unspliced precursors of *ndhA*, *petB*, *ycf3-int-1*, *petD*, *clpp1-int-1* accumulated to an increased level in the *pdm4* mutant (**Figure 6A**). The observation of altered intron processing was also confirmed by northern-blot analysis. The result indicated that unspliced precursors of *ycf3*, *petB*, *petD*, and *ndhA* were present and accumulated in a high level in *pdm4* and absent in the wild type. By contrast, the transcripts of *rps14* showed a higher efficiency of accumulation than the wild type (**Figure 6B**). Besides, RNA immunoprecipitation, followed by a quantitative PCR assay using the GFP antibody and *COM* plants, indicated that PDM4 was specifically associated with these target sequences in the *ndhA*, *petB*, *ycf3-1*, and *petD* transcripts (**Figure 6C**). The RNA immunoprecipitation efficiency was supported by western blot analysis (**Figure S5**), and the transcripts containing 18S rRNA were used as control.

### *pdm4* Mutant Is Defective in Chloroplast rRNA Accumulation

**Figure 7:**
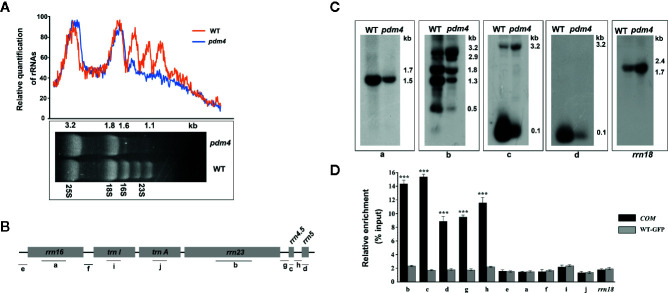
The chloroplast rRNA processing in *pdm4* mutant. **(A)** The contents of rRNAs in wild type and *pdm4*. 5 μg of total RNA from 3-week-old wild type and *pdm4* seedlings was separated by denaturing gel. 23S* is the breakdown product of the chloroplast 23S rRNA. rRNA was quantified using “ImageJ” software. **(B)** Diagram of the chloroplast rRNA operon and the locations of the probes (a–d) used for the RNA gel-blot analysis and used for RNA immunoprecipitation analysis (a**–**j). **(C)** RNA gel analysis of 16S rRNA (probe a), 23S rRNA (probes b), 4.5S rRNA (probe c), and 5S rRNA (probe d). The sizes of the transcripts (in kb) are shown. The 18S rRNA is shown as a loading control. **(D)** RNA immunoprecipitation analysis followed by a quantitative PCR assay. Probes b, d, c, a, i, f, and *rrn18* are fragments from the 23S rRNA, 5S rRNA, 4.5S rRNA, 16S rRNA, *trnI*, *trnA*, and 18S rRNA, respectively and probes e, f, g, h for the intergenic region. The asterisks indicate significant differences between WT-GFP and *COM* (Student’s t test; ***p < 0.001). Data are means (± SE) obtained from three replicates.

The chloroplast rRNAs, as well as two tRNAs, are arranged in one operon, and transcription depends on both PEP and NEP (Tiller and Bock, 2014). When denatured rRNA samples were separated on denaturing agarose gels, it was shown that the rRNA fragmentation pattern in the wild type was obviously different from *pdm4* mutant by using the ethidium bromide staining method (**Figure 7A**). The signal intensities of the 1.5 and 1.1-kb RNA corresponding to chloroplast 16S rRNA and a breakdown product of the chloroplast 23S rRNA were dramatically reduced in the *pdm4* mutant (**Figure 7A**).

To study the impaired accumulation of chloroplast rRNAs in detail, we performed northern-blot analyses to detect the 16S, 4.5S, 5S, and 23S rRNA by using four probes with an internal region (probes a, b, c, and d, respectively, as shown in **Figure 7B**). Upregulated levels of the 3.2-kb 23S–4.5S rRNA precursor were detected in the *pdm4* mutant, whereas the levels of the 0.12-kb 5S, 0.1-kb 4.5S, 0.5-kb 23S, and 1.5-kb 16S mature rRNAs were drastically downregulated (**Figure 7C**). We also have tested the interaction and/or association between PDM4 and its targets by RNA immunoprecipitation and qRT-PCR methods and using specific primers for transcripts containing the 23S, 5S, 4.5S, 16S rRNA, and two tRNAs, as well as the intergenic region (**Figure 7B**, probes e, f, g, h). We detected enrichment fragments of 23S, 5S, 4.5S rRNA, and the intergenic region in the anti-GFP immunoprecipitated, but not of 16S, *trnI*, *trnA*, and 18S rRNA (**Figure 7D**). Results from northern blots and RNA-Co-IPs support the conclusion that PDM4 associates with rRNA and affects rRNA processing. The RNA immunoprecipitation efficiency was also supported by western blot analysis (**Figure S5**).

## Discussion

Contrary to the increasing information about the subfamily classification and organellar targeting of PPR proteins in plants, the cellular role and function of most PPR proteins are still so far from fully being apprehended (Lee et al., 2019). Among these PPR proteins, several mutants show pigment defective or lethal phenotypes, such as *sel1* (Pyo et al., 2013), *ecd1* (Jiang et al., 2018), *ppr4*, *emb2654* (Lee et al., 2019), and *sot5/emb2279* (Huang et al., 2018) in addition to *pdm2* and *pdm3* mutants (Du et al., 2017; Zhang J. et al., 2017) which have been reported in our lab before. These works suggest that most functional PPR proteins located in the chloroplast mainly play a critical role in accumulation of chlorophyll and are essential for plant survival. In this study, we identified and characterized a novel PPR protein PDM4; the pigment defective phenotype of the mutant and complementation analysis with the *PDM4* gene demonstrated that PDM4 is indispensable for plant survival and growth.

Chlorophyll accumulation is a prerequisite for the maintenance of functional photosynthetic reaction centers and light-harvesting complexes (Pan et al., 2013). In the *pdm4*, the albino phenotype with a decrease of chlorophyll contents was observed (**Figure 1C**), indicating the photosystem complexes might be impaired, and in accordance with this hypothesis, the result of western blot analysis confirmed this possibility (**Figure 2E**). The chloroplast ultrastructure of *pdm4* exhibited an abnormal morphology, and the structure of the thylakoid membrane was seriously disturbed, suggesting that the lethal phenotype in these plants was probably attributed to developmental defects in chloroplasts. Furthermore, proper development and biogenesis of chloroplast play an essential role in the vast majority of important biological processes, including cell proliferation, secondary metabolite synthesis, and embryogenesis (Yin et al., 2012). Compared with our previous studies in *pdm2* and *pdm3* mutants, the development of homozygous embryo was also retarded especially after globular-to-heart stage (**Figures 3H–L**), suggesting that disruption of *PDM4* is the primary cause for embryogenesis defection in the *pdm4* mutant. Several PPR mutants have similar developmental defects during the embryogenesis process, *e.g.*, *Atppr2* (Lu et al., 2011), *fac19* (Yu et al., 2012), *At_dek36* (Wang et al., 2017), *blx* (Sun et al., 2018), and *ecd1* (Jiang et al., 2018). Borisjuk et al. (2005) suggested that the probable reason for these developmenal defects is energy source transformation from the endosperm to the chloroplast in seed development because chloroplast formed transiently and transformed into storage organelles during embryo development. This view also coincided with Barkan and Small (2014). Thus, blocking embryo development in *pdm4* mutant may result from the defective chloroplast by some indirect effects.

The expression of plastid-encoded genes of photosynthesis was closely linked with the developmental state of the chloroplast (Chi et al., 2008; Zhang Z. et al., 2017). qRT-PCR and northern-blot analysis showed that the levels of PEP-dependent transcripts were dramatically reduced; on the contrary, levels of NEP-dependent transcripts were obviously increased, suggesting that NEP functions correctly or efficiently in *pdm4* (**Figures 5A–D**). Compared with upregulated transcriptional level, the protein level of RpoB is significantly decreased in *pdm4* mutant (**Figure 5E**), and we obtained a similar result in the *pdm3* mutant (Zhang J. et al., 2017). It suggests that RpoB protein biosynthesis or stability is affected in the *pdm4*, leading to a dysfunctional PEP complex, just like the case that happened in the *pdm3* (Zhang J. et al., 2017). Thus, *PDM4* is necessary for an efficient and functional PEP transcription machinery. As to the reduced PEP activity, one possible reason is that PDM4 acts as a participant of nucleoid proteins, like PAPs or pTACs, or merely associates with them to participate in the regulation of PEP activity because *pdm4* mutant shares some similarities in molecular phenotypes with other PEP-related mutants (**Figures 5** and **6**; Zhang J. et al., 2017; He et al., 2019). As we know, pTACs always interacted with thylakoid membrane; we had not detected any signals with PDM4 in the thylakoid fragments (**Figure 4B**); this may indicate that there exists a weakly interaction between PDM4 and pTACs although none of the presented data show PEP/TAC-association or that PEP-activity is actually PDM4 dependent. Another explanation is that the stability of the transcripts may be decreased, or the rate of mRNA turnover is enhanced in those mutants. In fact, dozens of PPR proteins that affect the stability of chloroplast gene transcripts have been identified (Barkan and Small, 2014).

In higher plants, dozens of P-type PPR proteins are targeted to the chloroplasts and have been proved to be necessary in removing some distinct introns (Barkan and Small, 2014). For example, PPR5 was confirmed to promote the splicing of the group II intron within *rpl16* in Arabidopsis (Rojas et al., 2018); and in rice, AL2 collaborates a subset of chloroplast associated proteins to regulate splicing of both chloroplast group II and I introns (Liu et al., 2016). In this investigation, the disruption of *PDM4* affected the splicing of *ndhA*, *petB*, *clpP1-1*, *ycf3-1*, and *petD* transcripts, and to our surprise, *rps14* transcript accumulation was improved in this study (**Figure 6B**). This result does not fully coincide with *pdm3* in the respective transcript splicing events, *e.g.* the affected splicing sites are *ndhB*, *clpP1-1*, and *trnA*. Alternately, this probably suggests partially distinctive and redundant functions refer to PDM3 and PDM4 in the regulation of chloroplast gene expression. A recent work has shown that BFA2, a P-type PPR protein in the chloroplast, affects *atpF*-*atpA* transcript splicing by combining to the intergenic region of *atpF*-*atpA* and acts as a specific barrier to prevent *atpH/F* mRNA from exoribonuclease degradation (Zhang et al., 2019). Thus, we could not rule out the possibility that PDM4 acted as a barrier and resulted in a high efficiency in transcript accumulation of the *rps14* in *pdm4* mutant.

The result of agarose gel electrophoresis analysis validated that chloroplast rRNA was dramatically reduced in the *pdm4* mutant (**Figure 7A**). It is reported that PPR proteins are also involved in pre-rRNA processing and lead to reduced rRNA levels (Barkan and Small, 2014). To further assess this possibility, chloroplast rRNA was analyzed in detail by northern-blot analysis (**Figure 7C**). Compared with the wild type, 3.1-kb RNA representing the precursor of 23S and 4.5S rRNA was accumulated more in *pdm4* (**Figure 7C**). As a consequence of the increased precursor of rRNA in *pdm4*, we deduced that protein PDM4 was involved in the cleavage precursor of rRNA in the chloroplast, especially during the maturation of 50S rRNA. As pre-rRNA processing and ribosome assembly are intimately linked in the chloroplast, therefore, lesions in the ribosome assembly are frequently found in mutants with rRNA processing defects (Prikryl et al., 2008; Chi et al., 2011; Asakura et al., 2012). Coimmunoprecipitation assays produced evidence that PDM4 associates with rRNA and affects rRNA processing (**Figure 7D**), and we can also draw the conclusion that the PDM4 protein was specifically associated with these target sequences in the *ndhA*, *petB*, *ycf3-1*, and *petD* transcripts (**Figure 6C**). Thus, in *pdm4* mutant, the aberrant maturation and accumulation of chloroplast rRNA mutant may be due to a defect in ribosomal biogenesis/assembly. The *pmd4* mutant also showed an obvious downregulated level in the mature form of 16S rRNA, whereas an accumulation of precursor rRNA was not detected (**Figure 7C**). The decrease in mature 16S rRNA in *pdm4* appeared to be regarded as an indirect consequence of the defects in the 50S subunit biogenesis/assembly because PDM4 associated with the 30S particle was not found (**Figure 7**). This conclusion is further sustained by the identification of the RH22 in Arabidopsis (Chi et al., 2012).

PDM4 is required for PEP activity, polycistronic accumulation, and rRNA maturation. But it is hard to rule out which process is a dominant factor; because of the pleiotropic nature of knockout plants, it is particularly true when general plastid translation can be affected by the lack of ribosome processing and/or assembly, which indirectly results in dysfunctional transcription machine as the results of disrupted expression of the plastid encoded polymerase (PEP) and in turn affects RNA processing patterns and levels (Legen et al., 2002; Stoppel and Meurer, 2011). Furthermore, in *pdm4*, chloroplast development is severely damaged. We can find that PDM4 affecting the transcription level of PEP-dependent genes, RNA splicing of multiple chloroplast group II introns, and chloroplast rRNA accumulation. These processes influence and restrict each other, which together leads to the loss of chloroplast development. And based on our research, the key or the direct reason of this phenomenon is still not very clear. So, further study of PDM4 function should facilitates the general understanding the mechanism of plastid gene expression and chloroplast development.

## Data Availability Statement

All datasets generated for this study are included in the article/**Supplementary Material**.

## Author Contributions

JX designed the study. XW, LZ, YM, XL, LW, and JX performed the research. XW, XL, and JX analyzed the data. JX and XW wrote the paper. All authors discussed the results and made comments on the manuscript. All authors contributed to the article and approved the submitted version.

## Funding

This research was supported by the National Natural Science Foundation of China (grants. 31970653, 91954202 and 31670181).

## Conflict of Interest

The authors declare that the research was conducted in the absence of any commercial or financial relationships that could be construed as a potential conflict of interest.
