# Supplementary material for: PDM4, a Pentatricopeptide Repeat Protein, Affects Chloroplast Gene Expression and Chloroplast Development in Arabidopsis thaliana
Source: Front Plant Sci. 2020 Aug 11;11:1198. doi: 10.3389/fpls.2020.01198 (PMC7432182; doi:10.3389/fpls.2020.01198)
Supplement: Supplementary file 1 [file Table_1.docx]

| **Primers** | **Sequences (5’ to 3’)** | **Purpose** |
| --- | --- | --- |
| SALK_034168 T F  SALK_034168 T R | TCACTAACCAATAACACCACC  ATTGCTTGTGAGCCTTGGT | Mutant  identification |
| PDM4-RT-F  PDM4-RT-R | TGTCAGATGTGGCTGGCTTG  GCAGTATGTCCTTTCAGCCTC | RT-PCR |
| PDM4-PSN1301-F  PDM4-PSN1301-R | GCTCCTTCACCCGGGATCCATGAAGAGTGACTTCCTCACT  CCACCCTTTATCGGGATCCTACATCTTGTTCTTTACC | Transgene |
| PDM4-qRT-F  PDM4-qRT-R | TCAGGGTTGTATCTGGACG  CAGGGTGAGGTATGTGGAT | qRT-PCR |
| 23S-F  23S-R | AGATGGCGAGAGTCCAGTAG  GTCCTTGAACCGATAACCATC | Probe b |
| 4.5S-F  4.5S-R | GACGAGCCGTTTATCATTACGATAG  TTCAAGTCTACCGGTCTGTTAGG | Probe c |
| 5S-F  5S-R | TTCTGGTGTCCTAGGCGTAGAGGAA  TTTCCGCAGGACCTCCCCTACA | Probe d |
| 23S 3’F  23S 3’R | ATTCCGACTTCCCCAGAGCCTCC  TCTTGAATTCTCAAAACTTCTGT | Probe g |
| 4.5S 3’-F  4.5S 3’-R | CTTGTTCCTACATGACCTGATCA  TCGAACCATGAACGAAGAAAGGC | Probe h |
| 16S 5’-F  16S 5’-R | AACCCAATGAATGTGAGTTTTTC  TTCATAGTTGCATTACTTATAGC | Probe e |
| 16S-F  16S-R | CGCGTCTGATTAGCTAGTTGG  CCTTGCGGTTAAGGTAACGAC | Probe a |
| 16S 3’-F  16S 3’-R | TCAGGGAGAGCTAATGCTTCTTG  GTCGTGCGGGCCTCCTGCTGGGG | Probe f |
| trnI-F  trnI-R | GGGCTATTAGCTCAGTGGTAGAG  TGGGCCATCCTGGATTTGAACCA | Probe i |
| trnA-F  trnA-R | GGGGATATAGCTCAGTTGGTAGA  TGGAGATAAGCGGACTCGAACCG | Probe j |
| ndhA-F  ndhA-R | GAGGTCTATGGACTCATATG  TATTTCCTCTAGATGGACG | RNA immunoprecipitation |
| petB-F  petB-R | AATATGTTCCTCCGCATGTC  CGAAATACGTGCAGGATC | RNA immunoprecipitation |
| ycf3-F  ycf3-R | ATGTCGGCTCAATCTGAAG  GGTAATGACAGATCACAGC | RNA immunoprecipitation |
| petD-F  petD-R | GATCCTGTATTACGAGCTAA  CGCAGGTTCACCAATCAT | RNA immunoprecipitation |
| 18S-F  18S-R | GACTGTGAAACTGCGAATG  GCATCGTTTATGGTTGAGACT | Control |

RT-PCR., Reverse Transcription PCR; qRT-PCR., Quantitative Real-Time PCR
