# Supplementary material for: PDM4, a Pentatricopeptide Repeat Protein, Affects Chloroplast Gene Expression and Chloroplast Development in Arabidopsis thaliana
Source: Front Plant Sci. 2020 Aug 11;11:1198. doi: 10.3389/fpls.2020.01198 (PMC7432182; doi:10.3389/fpls.2020.01198)
Supplement: Supplementary file 2 [file Table_2.docx]

**FIGURE LEGEND**

**FIGURE S1** | **Early globular stage between WT and *pdm4.*** Cleared seeds observed under differential interference contrast (DIC). Embryo development of wild type **(A)** and *pdm4* **(B)** at early globular stage. Scale bars: 1 mm.

**FIGURE S2** | **PDM4 is a P-type PPR protein with 16 P repeat domains**. Schematic diagram of Arabidopsis PDM4 protein with a total of 16 PPR domains (P). Red arrow represents the T-DNA insertion mutation in *pdm4*.

**FIGURE S3** | **Amino acid sequence alignment of PDM4**. The amino acid sequence of PDM4 was aligned to homologous proteins from different species using ClustalW 2.0 (Larkin et al., 2007). The putative cleavage site of the transit peptide in PDM4 is indicated by an inverted triangle. Lines above the sequences show the predicted P- or PPR-like motifs.

**FIGURE S4** | **Phylogenetic tree of PDM4 and the 10 closest PPR family members from Figure S3.** Maximum likelihood (ML) tree was inferred with RAxML (version 7.2.8) using the PROTGAMMALGF model. The numbers on the branches refer to the bootstrap values (%) for 1000 replications and the scale bar at the bottom indicates units of amino acid substitutions per site. Complete deletion was adopted for the treatment of gaps and missing data.

**FIGURE S5 | The western blot analysis for detection of immunoprecipitation efficiency.** Immunoprecipitation followed by a western blot analysis by using the GFP antibody and *COM* plants in one blot, showing a relative enrichment and immunoprecipitation efficiency in output against the input and supernatant. The blot result of anti-ACTIN antibodies in WT and *COM* as control.

**TABLE S1** | **The sequences of primers used for PCR and RNA immunoprecipitation assays.**
