# Supplementary figures and images for: PDM4, a Pentatricopeptide Repeat Protein, Affects Chloroplast Gene Expression and Chloroplast Development in Arabidopsis thaliana
Source: Front Plant Sci. 2020 Aug 11;11:1198. doi: 10.3389/fpls.2020.01198 (PMC7432182; doi:10.3389/fpls.2020.01198)

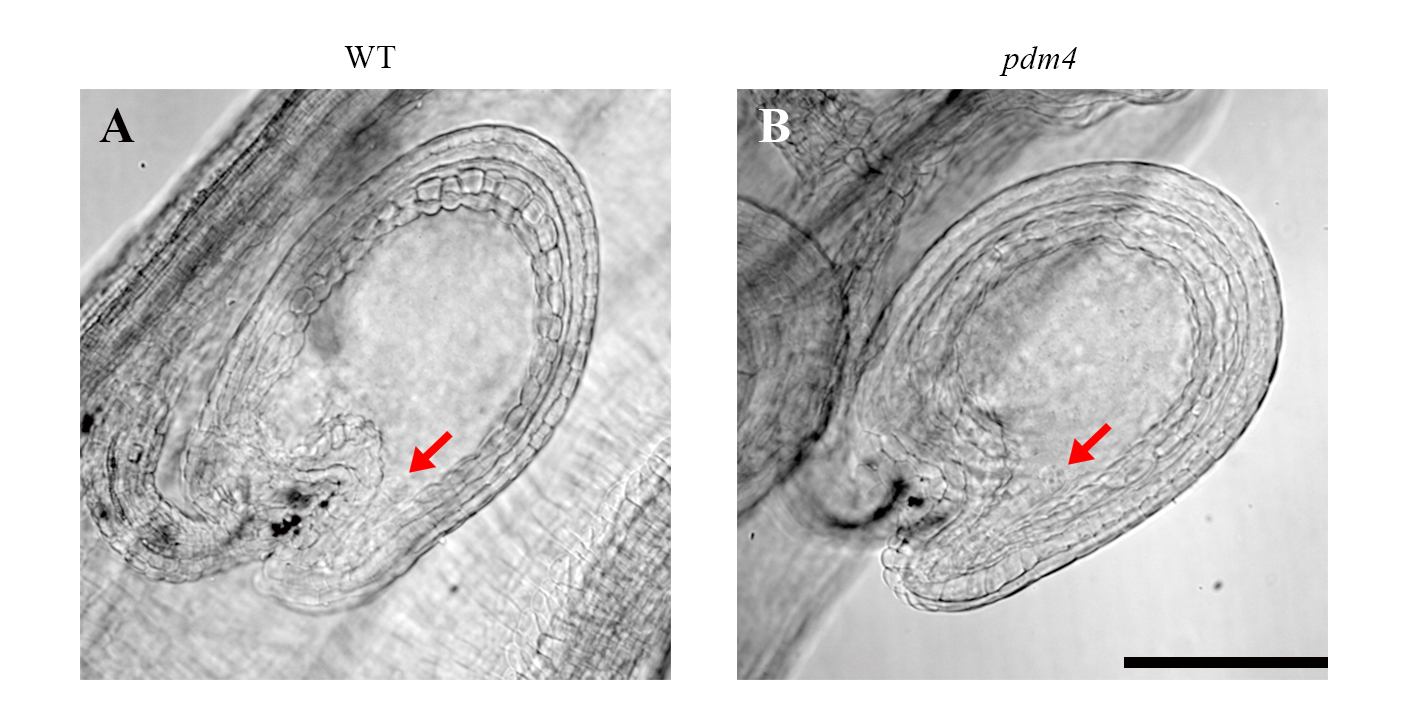

Supplement: Supplementary file 3 [file Image_1.jpeg]

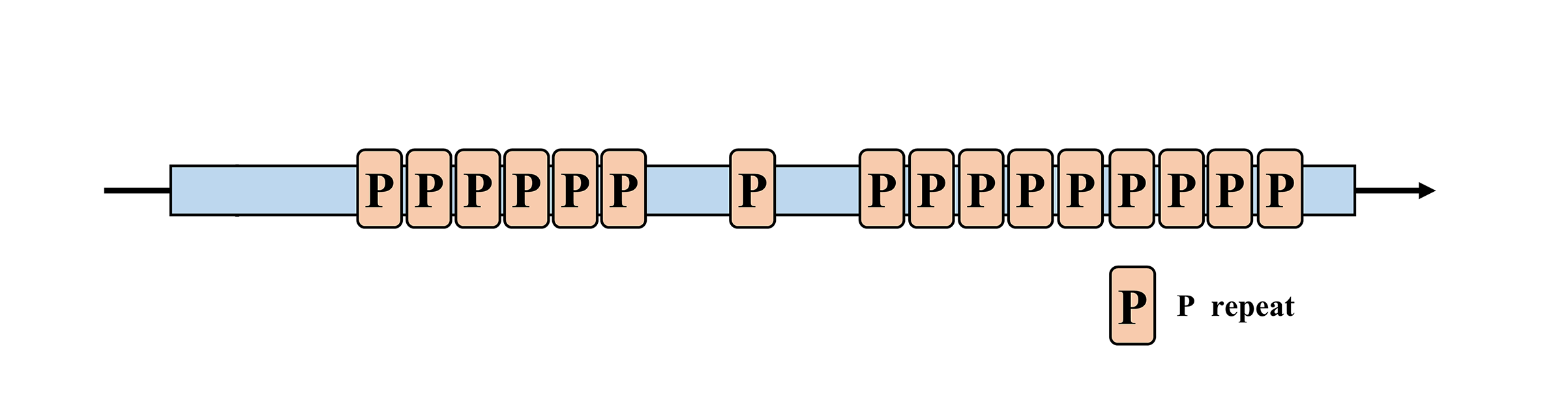

Supplement: Supplementary file 4 [file Image_2.tif]

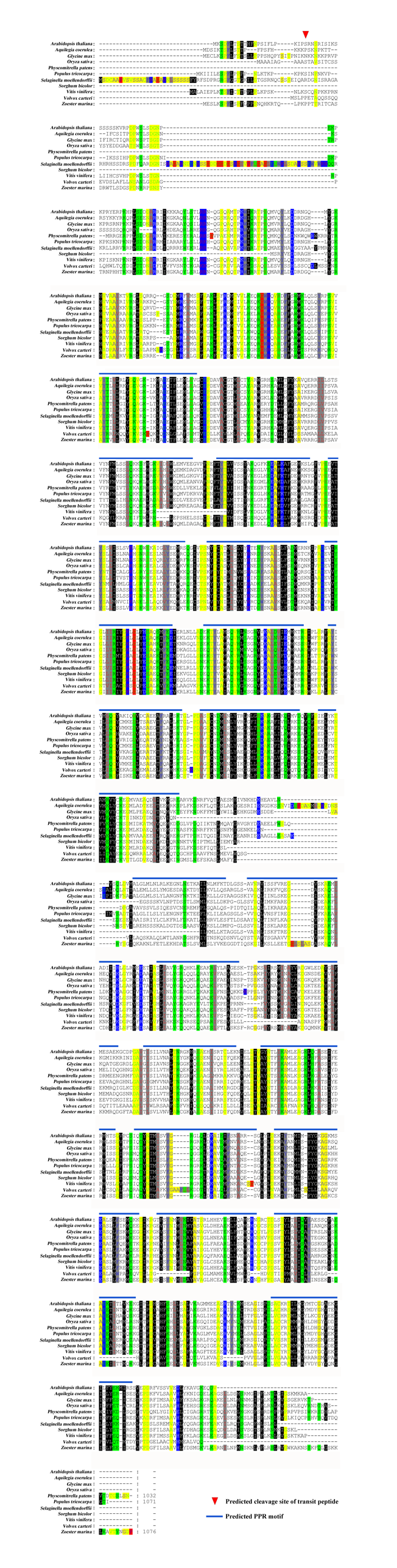

Supplement: Supplementary file 5 [file Image_3.tif]

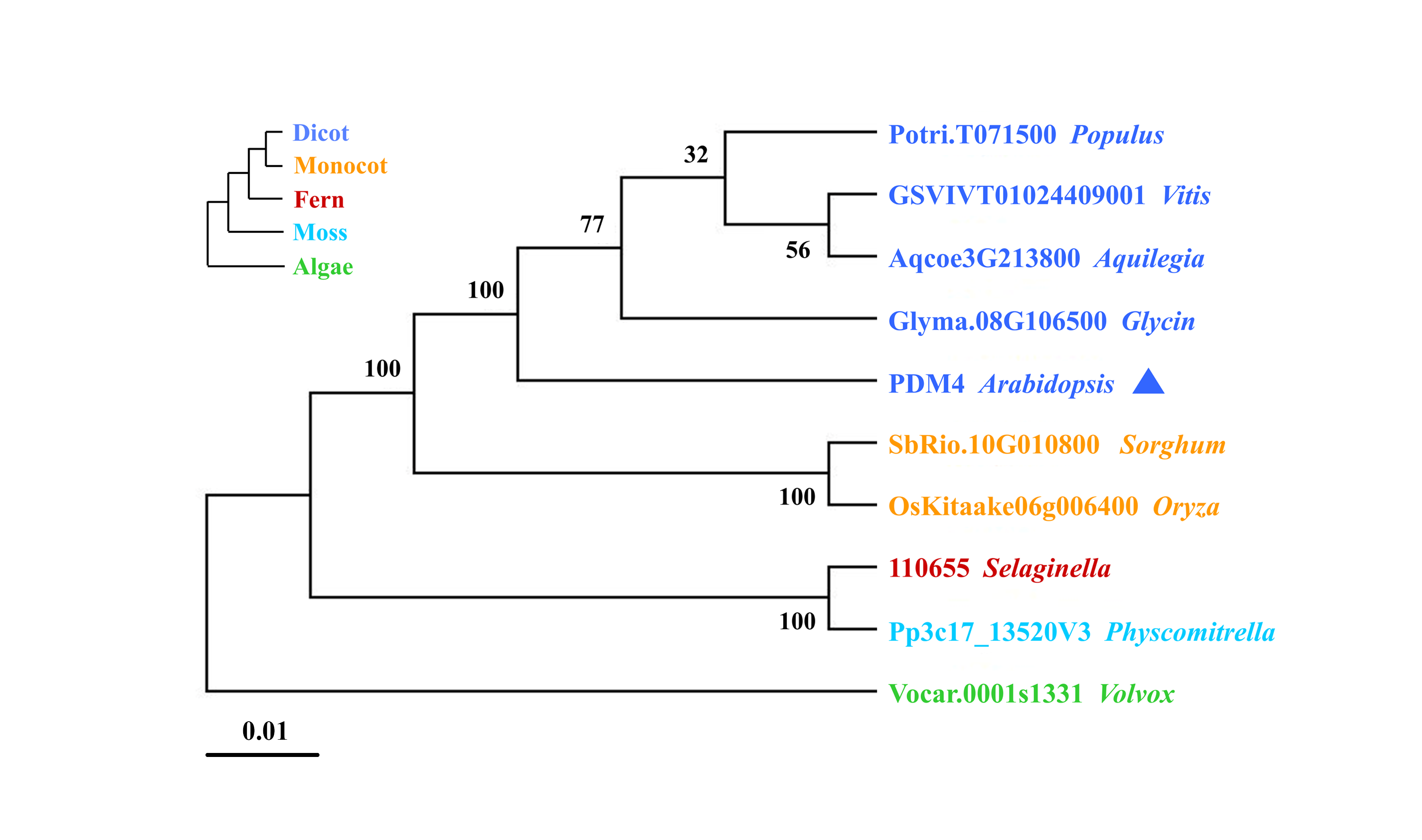

Supplement: Supplementary file 6 [file Image_4.tif]

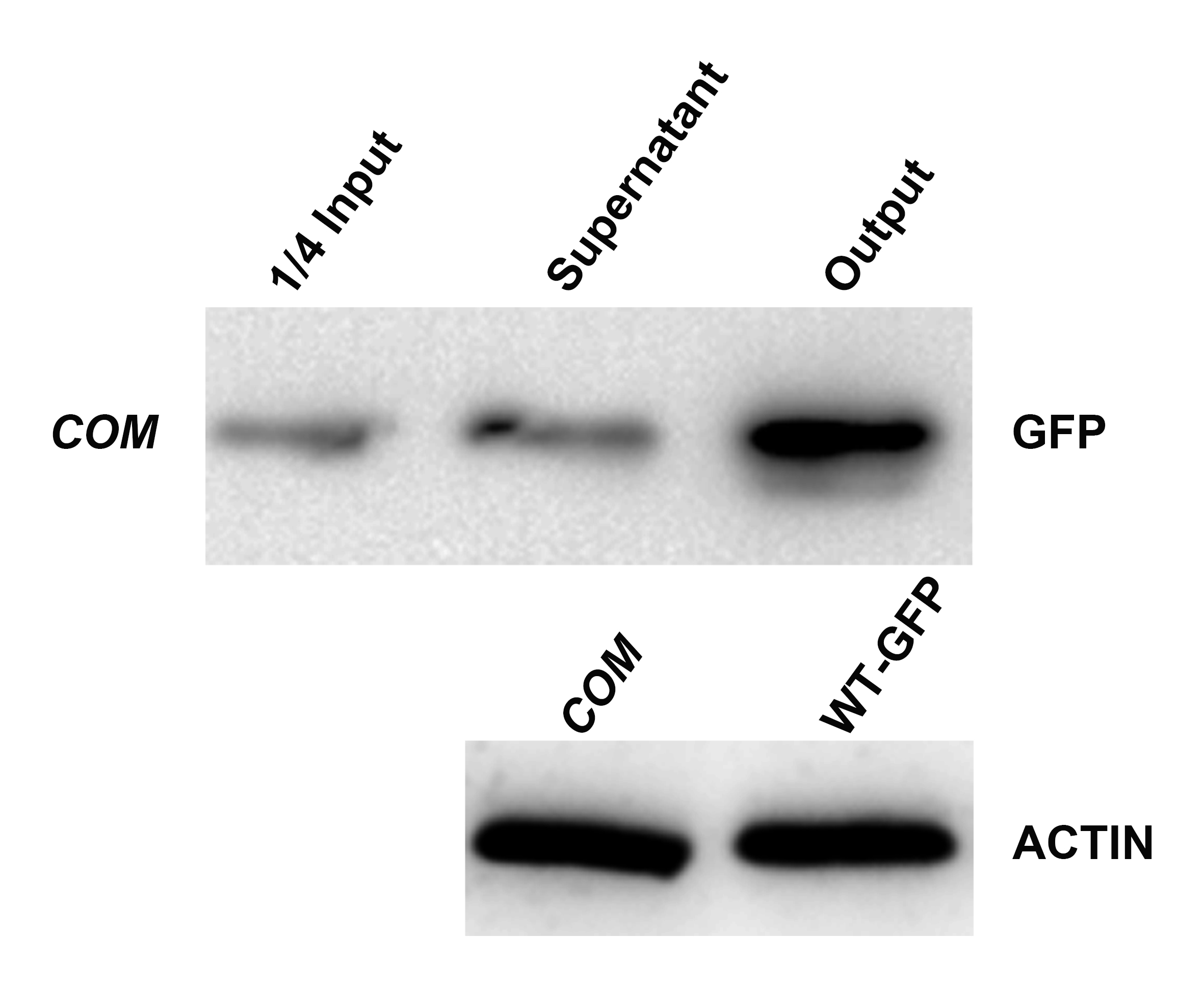

Supplement: Supplementary file 7 [file Image_5.jpeg]
